# Editorial: GPER: Control and Functions

**DOI:** 10.3389/fendo.2021.794344

**Published:** 2021-11-29

**Authors:** Yves Jacquot, Marilena Kampa, Sarah H. Lindsey

**Affiliations:** ^1^ CiTCoM, CNRS UMR 8038, INSERM U1268, Faculté de Pharmacie de Paris, Université de Paris, Paris, France; ^2^ Laboratory of Experimental Endocrinology, School of Medicine, University of Crete, Heraklion, Greece; ^3^ Department of Pharmacology, Tulane University, New Orleans, LA, United States

**Keywords:** G protein-coupled estrogen receptor, sexual dimorphism, central nervous system, heart tissue, signaling cascade, endocrine-disrupting chemicals, GPER turnover, modeling approaches

Since the pioneering work of Elwood V. Jensen (1920–2012), which led to the discovery of estrogen-binding “substances” shortly afterwards called estrophilin, the concept of estrogen receptor (ER) has evolved considerably (1–3). Initial reports localized ERs in the nuclear compartment of cells of reproductive tissues after a translocation process from the cytoplasmic membrane to promote transcription (4, 5). Until the cloning of ERβ in 1996 in rat prostate and ovary (6), only one receptor, named ERα, was known to bind the endogenous female hormone estradiol. In the following decades, at least three additional estrogen receptors were identified and cloned, i.e., GPER (7, 8), ERα46 (9), and ERα36 (10). ERα46 and 36 result from an alternative RNA splicing process of the gene *ESR1* encoding ERα (66 kDa), whereas GPER has its own transcript. The fact that estrogen receptors were discovered in the cytosol and cytoplasmic membrane of many different cell types, confirmed not only their ubiquitous character but also trafficking mechanisms in charge of the control of transcription. In the light of these observations, estrogen-mediated cellular signaling quickly became much more complex than initially claimed. In connection with these findings, two principal signaling processes were established, one initiated in the nucleus and the other at the cytoplasmic membrane.

Among estrogen receptors, GPER appears as the most atypical as it belongs to the family of class A (rhodopsin) G protein-coupled receptors (GPCRs) (11). Found in the cytoplasmic membrane, it can translocate to the membrane of the endoplasmic reticulum to exert specific functions (12) or to the trans-Golgi network for down-regulation (13). Based on what we know about the structure and functions of the classical estrogen receptor ERα, this discovery was extremely surprising and stimulated conflicting debates about the role of GPER, i.e., whether it directly binds estradiol or functions as a protein partner of ERα, similar to coactivators. While the latter scenario is not definitively excluded, depending on the context, a network of observations supporting the direct interaction of estradiol with GPER prompted its renaming from GPR30 (Luo and Liu). Since GPER binds the female hormone estradiol, one “basic” question is: does GPER play a role in sexual dimorphism? The answer is far from definitive, with sex differences in GPER distribution between males and females observed in some studies but not others (14). GPER-mediated sexual dimorphism may lie in providing differences between males and females in the social and behavioral network, as explained by Dovey and Vasudevan. In specific regions of the central nervous system (hypothalamus and amygdala), sex differences in the distribution of GPER impact synaptic plasticity and as such, the perception of anxiety, social and object recognition, and spatial memory (Kumar and Foster). In this regard, changes in the interaction of females with their environment during the estrous cycle could be explained, at least in part, by GPER expression fluctuations in the central nervous system during this same period, as explained by Llorente et al. Functional crosstalk with classical estrogen receptors (principally ERα and ER36) and tyrosine kinase receptors (principally EGFR) has also been established (15). As such, it is not surprising that GPER interferes with kinase cascades and calcium flux, with consequences in the cardiovascular system, as explained by Tran, as well as on cell growth and neuronal transmission (Kumar and Foster). In this regard, it should be stressed that the submembrane part of GPER encompasses four Ca^2+^-calmodulin-binding sites, an observation that contributes to making this protein atypical (16). Such mechanisms could also play a role in glucose metabolism and obesity, opening new and exciting clinical opportunities.

As observed with the classical estrogen receptor ERα, endocrine-disrupting chemicals such as bisphenols, dichlorodiphenyltrichloroethane (DDT), polychlorinated biphenyls (PCBs) and phytoestrogens (e.g., genistein) promote cell proliferation and migration through GPER, as reviewed by Périan and Vanacker. Such observations impose the development of a low-to-middle throughput method to detect endocrine disrupting agents acting through GPER. Such method is now available (Périan et al.). In this context, an impact of soy isoflavones on promoting glial cell migration through GPER has been evidenced (Ariyani et al.). Strikingly, tamoxifen, which is widely used to fight estrogen-dependent breast cancer by directly interfering with the estradiol-binding site of ERα, up-regulates GPER and enhances cell proliferation, an observation that could explain, at least in part, tamoxifen resistance, as highlighted by Molina et al.

Hence, GPER appears not only as a key pleiotropic actor of mammalian hormone homeostasis but also as a promising target for the modulation of related physiological and pathological actions. However, the lack of crystal structure for GPER remains an obstacle to the development of modulators. Computational (virtual) approaches consisting of multiple protein sequence alignment combined with molecular docking of compound libraries have been proposed to identify new potential modulators or model explaining the mode of binding of active molecules (Grande et al.).

In this Research Topic celebrating 25 years since the discovery of GPER, many aspects of the functional role of GPER will be discussed.

## Author Contributions

All authors have contributed to the article and have approved the submitted version.

## Funding

This work was supported by National Institutes of Health grant number HL133619 (SHL) and by the German Academic Exchange Service (DAAD project-ID: 57515112 (MK).

## Conflict of Interest

The authors declare that the research was conducted in the absence of any commercial or financial relationships that could be construed as a potential conflict of interest.

## Publisher’s Note

All claims expressed in this article are solely those of the authors and do not necessarily represent those of their affiliated organizations, or those of the publisher, the editors and the reviewers. Any product that may be evaluated in this article, or claim that may be made by its manufacturer, is not guaranteed or endorsed by the publisher.
